# Whole genome and transcriptome analysis reveal adaptive strategies and pathogenesis of *Calonectria pseudoreteaudii* to *Eucalyptus*

**DOI:** 10.1186/s12864-018-4739-1

**Published:** 2018-05-10

**Authors:** Xiaozhen Ye, Zhenhui Zhong, Hongyi Liu, Lianyu Lin, Mengmeng Guo, Wenshuo Guo, Zonghua Wang, Qinghua Zhang, Lizhen Feng, Guodong Lu, Feiping Zhang, Quanzhu Chen

**Affiliations:** 1 Jinshan College, Fujian Agriculture and Forestry University, Fuzhou, 350002 China; 2 Forestry College, Fujian Agriculture and Forestry University, Fuzhou, 350002 China; 3 State Key Laboratory of Ecological Pest Control for Fujian and Taiwan Crops, Fujian Agriculture and Forestry University, Fuzhou, 350002 China

**Keywords:** *Eucalyptus*, *Calonectria* leaf blight, Detoxification, Secondary metabolism, Cell wall degrading enzymes

## Abstract

**Background:**

Leaf blight caused by *Calonectria* spp. is one of the most destructive diseases to affect *Eucalyptus* nurseries and plantations. These pathogens mainly attack *Eucalyptus*, a tree with a diversity of secondary metabolites employed as defense-related phytoalexins. To unravel the fungal adaptive mechanisms to various phytoalexins, we examined the genome of *C. pseudoreteaudii*, which is one of the most aggressive pathogens in southeast Asia.

**Results:**

A 63.7 Mb genome with 14,355 coding genes of *C. pseudoreteaudii* were assembled. Genomic comparisons identified 1785 species-specific gene families in *C. pseudoreteaudii*. Most of them were not annotated and those annotated genes were enriched in peptidase activity, pathogenesis, oxidoreductase activity, etc. RNA-seq showed that 4425 genes were differentially expressed on the eucalyptus(the resistant cultivar *E. grandis*×*E.camaldulensis* M1) tissue induced medium. The annotation of GO term and KEGG pathway indicated that some of the differential expression genes were involved in detoxification and transportation, such as genes encoding ABC transporters, degrading enzymes of aromatic compounds and so on.

**Conclusions:**

Potential genomic determinants of phytoalexin detoxification were identified in *C. pseudoreteaudii* by comparison with 13 other fungi. This pathogen seems to employ membrane transporters and degradation enzymes to detoxify *Eucalyptus* phytoalexins. Remarkably, the *Calonectria* genome possesses a surprising number of secondary metabolism backbone enzyme genes involving toxin biosynthesis. It is also especially suited for cutin and lignin degradation. This indicates that toxin and cell wall degrading enzymes may act important roles in the establishment of *Calonectria* leaf blight. This study provides further understanding on the mechanism of pathogenesis in *Calonectria*.

**Electronic supplementary material:**

The online version of this article (10.1186/s12864-018-4739-1) contains supplementary material, which is available to authorized users.

## Background

**Fig. 1:**
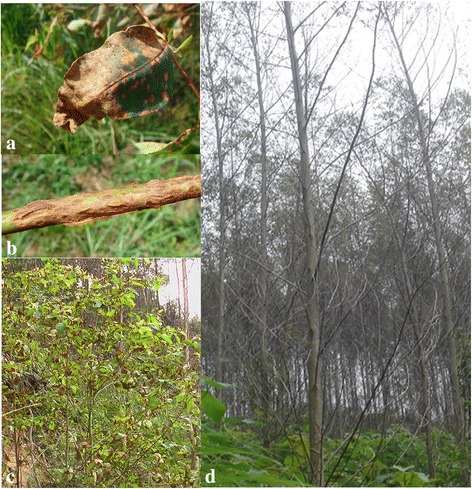
Infection of *C. pseudoreteaudii* on *Eucalyptus* tree. **a**-**c**. Symptoms of *C. pseudoreteaudii* on *Eucalyptus* leaf and twigs, including leaf blight and stem cankers. **d**. Defoliated *Eucalyptus* trees caused by *C. pseudoreteaudii* in a plantation

The genus *Calonectria* (anamorph state: *Cylindrocladium*) includes a group of pathogens commonly found in tropical and sub-tropical regions [1–3]. They can infect more than 335 plant species, causing serious economic losses in forestry, agricultural and horticultural crops [4–7]. *Eucalyptus* species are among the main hosts of these pathogens, as they can attack *Eucalyptus* leaf, stem, and branch tissues (Fig. 1a-c), establishing CLBs, stem cancer and cutting rot [8, 9]. Of these, CLBs are the most devastating diseases in *Eucalyptus* nurseries and plantations [10–12].

The *Calonectria* genus comprises at least 68 species that are further classified into 13 groups according to the morphological features and DNA sequences [3, 13]. *C. reteaudii* complex are the causal agents of CLBs in Australia, South America and Southeast Asia [14]. This complex currently includes six described species, including *C. microconidialis, C. pentaseptata, C. pseudoreteaudii, C. queenslandica, C. reteaudii and C. terrae-reginae* [12, 13]. They share a common feature in that their anamorphs *Cylindrocladium* all have a clavate vesicle with multiseptate macroconidia. Of these species, *C. pseudoreteaudii* is the first species of this genus found in Fujian province, China. It is also one of the most widely-distributed and aggressive species in this region [15]. *C. pseudoreteaudii* infects *Eucalyptus* tissue mainly by conidia [16]. Infected leaf symptoms begin with water-soaked lesions, which rapidly develop into extensive tissue maceration and necrosis under high humidity condition, resulting in leaf blotch and shoot blight and leading to serious defoliation and eventually death (Fig. 1d). It is estimated that annual economic losses due to this disease are over $7.8 million in Fujian alone [17].

Recently, whole genome sequencing has been employed in the study of plant pathogenic fungi [18]. This technology with the application of comparative genomics analysis has accelerated the study of plant pathogens and significantly advanced our understanding of different pathogens [19–21]. To date, over 100 plant pathogenic fungi and oomycetes have completed genome sequencing. The results indicate remarkable diversity in genome size and architecture of pathogens with various ecological niches and lifestyles [22, 23]. Some pathogens tend to evolve smaller genomes than their free-living relatives, while others exhibit a trend towards larger genomes by increasing repetitive DNA. Variations in genome size are always accompanied by expansions and contractions of specific gene families [22, 24, 25]. Obligate biotrophic pathogens such as rust fungi, powdery mildews, and downy mildews have a reduced set of genes encoding plant cell wall hydrolases. Necrotrophic and hemibiotrophic pathogens seem to expand gene families involved in plant cell wall degradation and secondary metabolism [26, 27]. Moreover, phytopathogenic fungi always evolve an appropriate genome to adapt to specific ecological niches. For example, *Ustilaginoidea virens* has an adaptation to occupy host florets by reducing gene inventories for polysaccharide degradation, nutrient uptake, and secondary metabolism [28].

In classification, *Calonectria* belongs to Nectriaceae family. So far, many species of this family have completed genome sequences, for example, *Fusarium* spp. and *Neonectria ditissima* [29–32]. Genomic analysis of *Nectria haematococca* (*F. solani*) indicated three supernumerary chromosomes which could account for individual isolates having different environmental niches [33]. Comparative genomics of *F. oxysporum* with other *Fusarium* spp. revealed LSGR rich in transposons and genes related to pathogenicity [34]. This entire LSGR can transfer between strains of *F. oxysporum*, and convert a non-pathogenic strain into a pathogen [35]. However, an investigation has not been performed on the genome of *C. pseudoreteaudii*, or other *Calonectria* species.

Given the economic importance of *Eucalyptus*, we sequenced the genome of *C. pseudoreteaudii* and analyzed its transcriptome cultured on *Eucalyptus* tissue medium and PDB, respectively. This can promote our understanding on the pathogenicity mechanism and provide reference for developing effective disease management strategies.

## Results and discussion

### Genome sequencing and general features

The genome of *C. pseudoreteaudii* YA51 was sequenced using Illumina Hiseq sequencing platform. The total reads were 13,584 Mb in length, representing an approximate 213-fold sequence coverage (Additional file 1: Table S1). A 63.57 Mb draft genome was assembled with 507 scaffolds (>500 bp; Table 1). Scaffold N50 is 1.32 Mb and the largest scaffold is 5.15 Mb. CEGMA analysis indicated that 240 out of 248 (96.7%) core eukaryotic genes were identified in the *C. pseudoreteaudii* genome. This suggests a high degree of completeness for the *C. pseudoreteaudii* genome assembly. The estimated proportion of repeat sequences in *C. pseudoreteaudii* is 9.26% (Additional file 1: Table S2). Most of these repetitive sequences (91.8%) are TEs. Similar to other fungi, the *C. pseudoreteaudii* genome includes a large proportion Gypsy and Copia retrotransposons.

**Table 1:** The general features of *C. pseudoreteaudii*

Feature	*C. pseudoreteaudii*
Assembly size (Mb)	63.7
Coverage (fold)	213.2
Scaffold number	507
N50 scaffold (kb)	1316
N90 scaffold (kb)	261
GC content(%)	48.3
Repeat rate (%)	9.27
Gene number	14,355
Gene density (genes· Mb^−1^)	225
Average gene length (bp)	1514.8
Number of exons	40,210
Average number of exons per gene	2.8
Average exon size (bp)	487.6
Number of Introns	25,855
Average number of intron per gene	1.8
Average intron size (bp)	82.7
tRNA genes	370
Secreted proteins	1178
SSCRPs	207
Genbank accession number	MOCD00000000

A total of 14,355 genes including 1178 secreted protein genes were predicted from the annotated *C. pseudoreteaudii* genome, 87% of which were supported by RNA-seq data. The coding capacities were similar to those of other ascomycetes such as *F. solani* and *N. ditissima* [22, 36]. Among these proteins, 11,636 (81.05%) were similar to the sequences in NCBI, 4298 (29.94%) were mapped to the KEGG database, 11,760 (81.92%) were classified in the NOG database (Additional file 2: Figure S1), and 8972 (62.5%) were assigned to GO terms (Additional file 2: Figure S2).

### Phylogeny and analysis of gene families

**Fig. 2:**
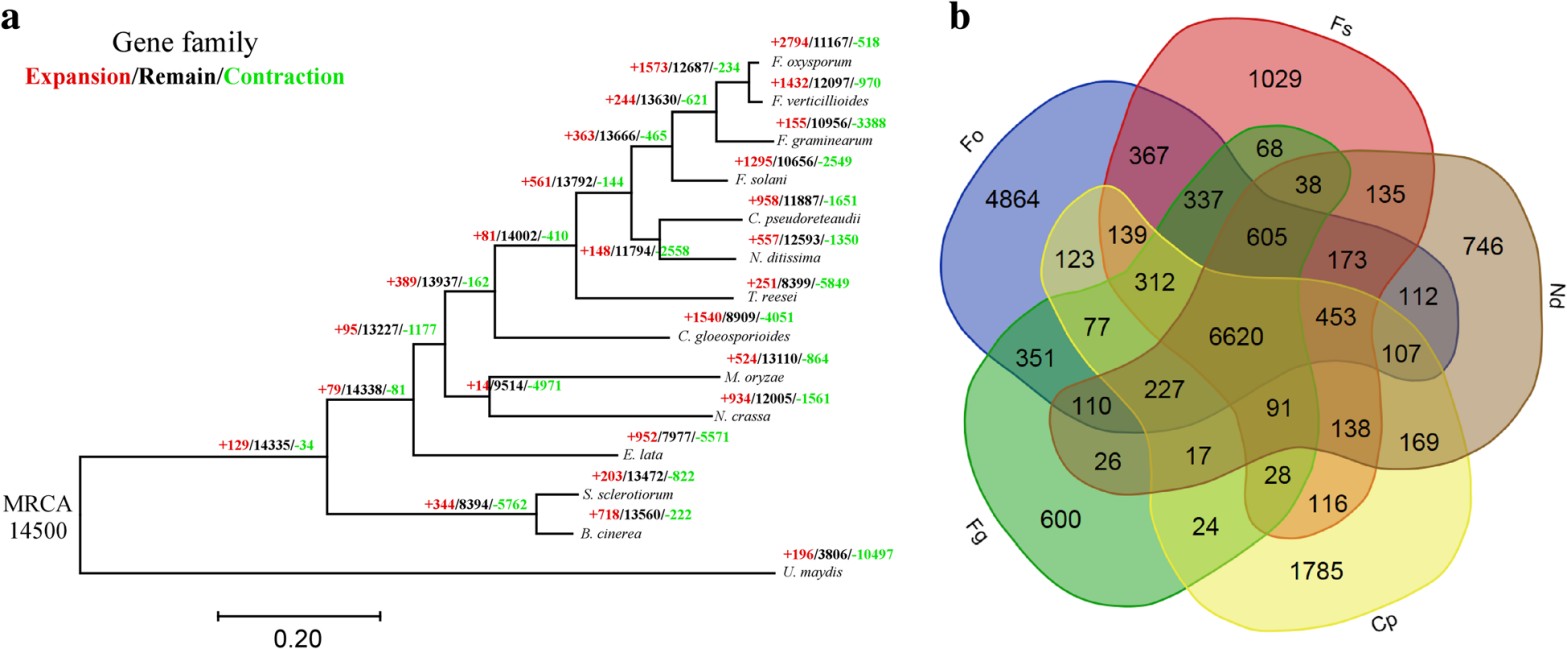
Phylogenetic relationship and analysis of gene families. **a**. Phylogenetic analysis of *C. pseudoreteaudii* and 13 other ascomycota fungi. Predicted pattern of gain and loss of gene families in 14 organisms used in this study. The numbers on the branches of the phylogenetic tree correspond to acquired (left, red), conserved (middle, black), lost (right, green), by comparison with the putative pan-proteome. **b**. The numbers represent counts of gene families which are either specific to each species or common shared among multiple species. Cp, *C. pseudoreteaudii*; Nd, *Neonectria ditissima*; Fs, *Fusarium solani*; Fg, *F. graminearum*; Fo, *F. oxysporum*; Fv, *F. verticillioides*

The phylogenetic position of *C. pseudoreteaudii* was evaluated among 13 other fungal species (12 ascomycota and one basidiomycota outgroup) using 1032 highly conserved single-copy orthologous genes. Compared with other Nectriaceae fungi, *C. pseudoreteaudii* was more closely related to *N. ditissima*, which is an important pathogen on apples (Fig. 2a). We have identified 14,500 gene families in 14 organisms. More common gene families (169) shared by *C. pseudoreteaudii* and *N. ditissima* suggesting that these two relatives retained more common characteristics (Fig. 2b). In addition, there were 1785 species-specific gene families (including 1828 genes) in *C. pseudoreteaudii*. However, most of them were not annotated due to a lack of homology with proteins in the pfam database. We speculate that these genes may have recently formed to adapt to a specific host. Those annotated species-specific genes were enriched in several functional items (Additional file 2: Figure S3): peptidase activity, pathogenesis, oxidoreductase activity, etc.

**Fig. 3:**
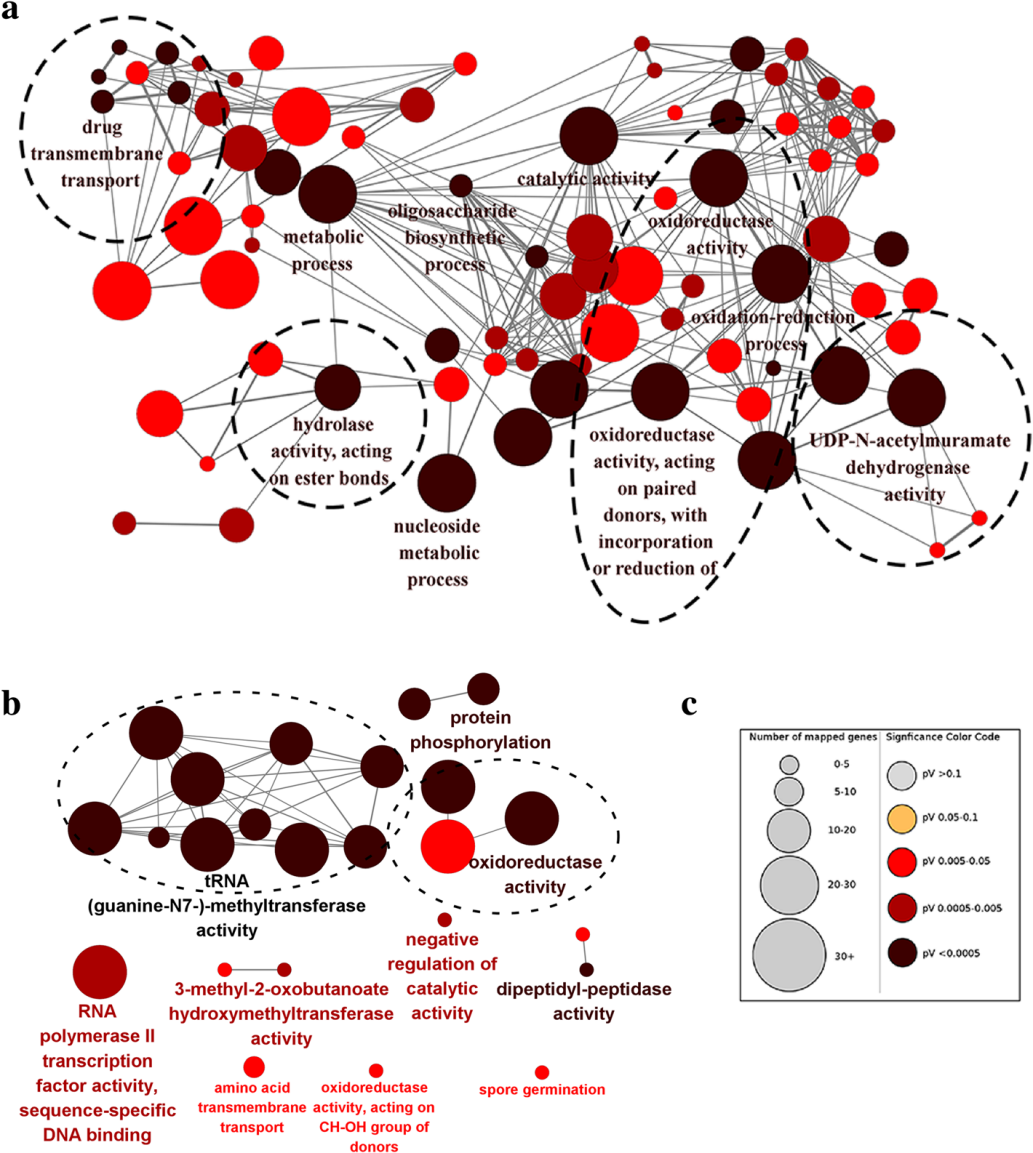
GO analysis of the expanded and contracted gene families in *C. pseudoreteaudii*. **a** The expanded gene families were significantly enriched in oxidation-reduction processes, drug transmembrane transport, hydrolase activity and UDP-N-acetylmuramate dehydrogenase activity. **b** The contracted gene families were significantly enriched in tRNA methyltransferase activity, protein phosphorylation, and oxidoreductase activity. **c** Each circle represents a significantly enriched GO term (*P* < 0.05, hypergeometric test, Bonferroni step-down correction). The color code reflects *P* values and the circle size indicates the number of genes relative to each GO term

Compared with *N. ditissima*, 958 and 1651 gene families have been predicted to have experienced expansion or contraction in *C. pseudoreteaudii*, respectively (Fig. 2a). The GO analysis of significantly expanded and contracted gene families are showed in Fig. 3 (*P* < 0.01). The contracted gene families are functionally classified into tRNA methyltransferase activity, protein phosphorylation and oxidoreductase activity (Fig. 3b). Most of the expanded gene families are functionally classified into oxidation-reduction processes, suggesting a crucial role in the host adaptive process (Fig. 3a). Furthermore, three families with 16 members are related to cellular aromatic compound metabolic processes. 7 genes were involved in drug transmembrane transport. There were also some expanded genes related to hydrolase activity and UDP-N-acetylmuramate dehydrogenase activity (Additional file 2: Figure S4). We suggested that genes in these functional categories may play crucial role in the ecological adaptation of *C. pseudoreteaudii*.

### Transcriptome analyses

To identify genes and pathways that may involve in pathogenesis of *C. pseudoreteaudii* to *E. grandis*×*E.camaldulensis* M1, differentially expressed genes of *C. pseudoreteaudii* cultured on the eucalyptus (*E. grandis*×*E.camaldulensis* M1) tissue induced medium were analyzed with *C. pseudoreteaudii* cultured on the PDB medium as control.

**Fig. 4:**
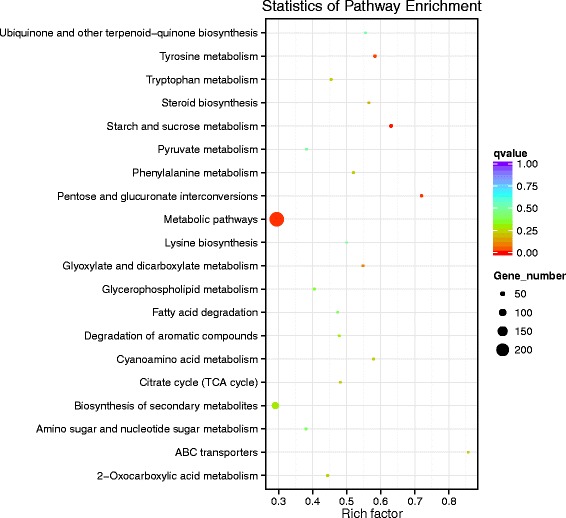
KEGG pathway analysis of differentlly expressed genes of *C. pseudoreteaudii* in *Eucalyptus* tissue medium culture. The differentlly expressed genes (log2 fold-changes) were significantly enriched in ABC transporters, pentose and glucuronate interconversions, starch and sucrose metabolism, tyrosine metabolism, degradation of aromatic compounds and so on

Totally, 33.05 Gb of sequence data were generated from 6 samples. 85% of the reads could be located on the genome of *C. pseudoreteaudii*. With *P* value < 0.05 and log2(fold change) ≥1 as the parameter, 1726 and 2699 genes were found to up-regulate and down-regulate on induced medium, respectively. To provide a general view on the functions and processes, differentially expressed genes were annotated in GO term and KEGG pathways. The result indicated that there were several significantly enriched terms of up-regulated genes including transporter activity, polygalacturonase activity, transcription factor activity, copper ion transmembrane transporter activity, oxidoreductase activity, etc. (Additional file 2: Figure S5a). While there were no significantly terms of down-regulated genes (Additional file 2: Figure S5b). KEGG pathway analysis showed that these differentially expressed genes were involved in ABC transporters, pentose and glucuronate interconversions, starch and sucrose metabolism, tyrosine metabolism, degradation of aromatic compounds and so on (Fig. 4). The more down-regulated genes suggested that the cultivar eucalyptus tissue caused stress to the growth of *C. pseudoreteaudii*. Previous research has indicated that polyphenols and flavonoids were important defensive compounds on the resistance of *Eucalyptus* to *Calonectira* [37]. Thus, these defensive compounds could be one source of the growth stress of *C. pseudoreteaudii* in induced medium. While *C. pseudoreteaudii* could clear defensive compounds from host by degradation, or segregate them by transporter, then relieve the growth stress from host.

### Genes involved in secondary metabolism are remarkably expanded in *C. pseudoreteaudii*

Filamentous fungi produce a diverse array of secondary metabolites during their development. Phytopathogens employ secondary metabolites as weapons to facilitate the invasion and colonization, including polyketides, nonribosomal peptides, terpenes, etc. [38, 39]. Backbone enzymes are primarily responsible for the synthesis of these metabolites. In this study, we identified 57 backbone enzyme genes in the genome of *C. pseudoreteaudii*, including 25 PKS, 26 NRPS, and 2 DMAT (Table 2, Additional file 1: Table S3), which was more than the average level for ascomycete [26]. It suggested a great production capacity of secondary metabolites in *C. pseudoreteaudii*. RNA-seq showed that seven of these were up-regulated in mycelia of *Eucalyptus* tissue medium culture, including three NPRS and four PKS genes. One PKS gene (Cp_Cap07289), annotated as a putative conidial pigment polyketide synthase, was up-regulated during infection with *Eucalyptus* leaves.

**Table 2:** The backbone genes responsible for the biosynthesis of secondary metabolites

Species	NRPS^a^	PKS^b^	HYBRID^c^	DMAT^d^	Total
*Calonectria pseudoreteaudii*	26	25	4	2	57
*Fusarium graminearum*	21	15	1	0	37
*Magnaporthe oryzae*	11	15	3	3	32
*Sclerotinia sclerotiorum*	10	18	0	1	29
*Neurospora crassa*	6	8	0	1	15
*Trichoderma reesei*	13	12	2	0	27

^a^NRPS denotes nonribosomal peptide synthases; ^b^PKS denotes polyketide synthases; ^c^HYBRID denotes NRPS-PKS; ^d^DMAT dimethylallyl tryptophan synthases

The biosynthesis of secondary metabolites also requires modifying enzymes, such as dehydrogenases, methyl-transferases, and CYPs [40]. CYP enzymes catalyze the conversion of hydrophobic intermediates from primary and secondary metabolic pathways and detoxify natural and synthetic antifungal compounds, allowing fungi to grow under different conditions [41]. A total of 161 CYPs were identified in the genome of *C. pseudoreteaudii*. 20 of which were up-regulated in *Eucalyptus* tissue medium culture and nine were unique to *C. pseudoreteaudii*.

The backbone enzymes genes of secondary metabolism, and other related genes including modified enzyme genes, regulatory genes and transporter genes, are typically closely clustered in the genome [42, 43]. Therefore, it is important to identify secondary metabolism gene cluster. 35 gene clusters involved in secondary metabolism were found in the genome of *C. pseudoreteaudii* (Additional file 1: Table S4). 22% of which contained at least one transporter. Overall ten CYP genes were found within these gene clusters.

### Transport capacity is enhanced in *C. pseudoreteaudii*

**Fig. 5:**
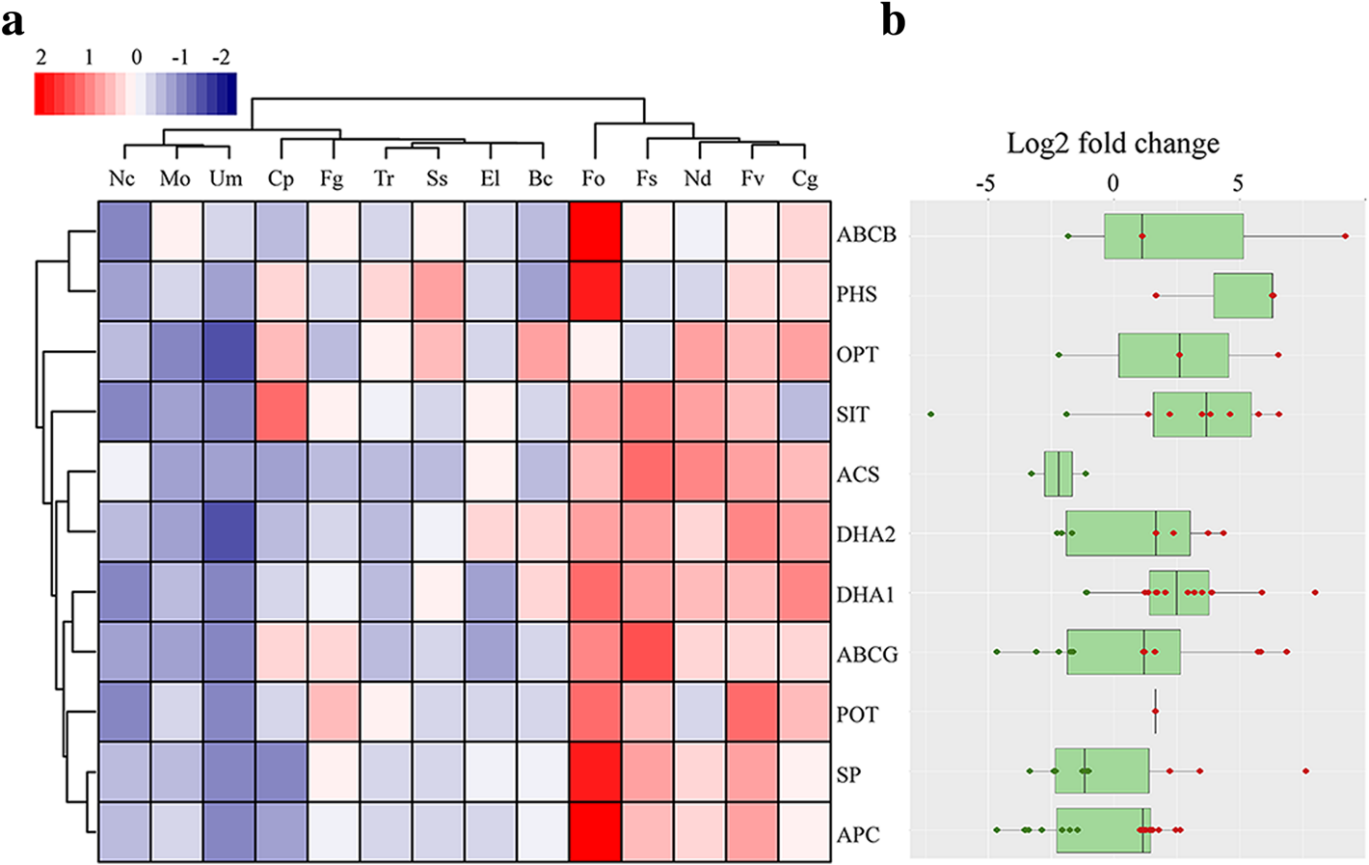
Comparison and expression patterns of membrane transporter families in *C. pseudoreteaudii* and 13 ascomycota fungi. **a**. Hierarchical clustering of membrane transporter families from *C. pseudoreteaudii* and 13 fungal genomes. Tr, *Trichoderma reesei*; El, *Eutypa lata*; Cg, *Colletotrichum graminicola*; Nc, *Neurospora crassa*; Mo, *Magnaporthe oryzae*; Ss, *Sclerotinia sclerotiorum*; Bc, *Botrytis cinerea*; Ud, *Ustilago maydis*. Transporter families are represented by their family names according to the Transporter Collection Database (www.tcdb.org). Overrepresented (pink to red) and underrepresented (gray to blue) domains are depicted as Z-scores for each family. Approximately unbiased (AU) *P*-values (%) are computed by 1000 bootstrap resamplings by using the R package pvclust. **b**. Box-plot of gene expression of members in each membrane transporter family of *C. pseudoreteaudii* in *Eucalyptus* tissue medium culture (log2 fold-changes). The Tukey whiskers indicate 1.5 times the interquartile range from the 25th and 75th percentiles

Membrane transporters can function in the transport of nutrients and removal of toxic compounds. We identified 679 membrane transporter genes in the *C. pseudoreteaudii* genome (Additional file 1: Table S5), which is about the same as *F. solani* and *F. verticillioides*. However, *C. pseudoreteaudii* contains more ABC transporters compared with the 13 other fungi. ABC transporter is a virulence factor that increases tolerance of the pathogen by extruding the natural and synthetic toxins from the cell. Several subfamilies of MFS transporters expanded in the genome of *C. pseudoreteaudii*, such as SITs (Fig. 5a). SITs can help pathogens to overcome iron limitations, and enhance pathogenicity [44, 45]. This suggests that ABC and MFS transporters may play a role in *C. pseudoreteaudii* during adaptation to the specific niche.

RNA-seq data showed that 105 membrane transporters were significantly up-regulated on the *Eucalyptus* tissue medium culture (Additional file 1: Table S6). Interestingly, most of them were MFS transporters including SITs and DHA1 (Fig. 5b). DHA1 is also reported to transport specific drugs and confer multidrug resistance of pathogens [46]. These results further illuminated that this pathogen possibly enhanced transport capability to colonize a host niche that is enriched in antifungal compounds.

### *C. pseudoreteaudii* genome is suited for cutin and lignin degradation

The plant cuticle is the outermost defense against pathogens. In a previous study, the cuticle thickness is a key factor for *Eucalyptus* against *Calonectria* [47]. However, the production of cutinase in the early infection can facilitate the penetration of the cuticle [48]. Remarkably, the *C. pseudoreteaudii* genome contains more cutinase genes than most of the fungi in this study (Additional file 1: Table S7), suggesting an enhanced potential for cuticle degradation. Cutinase genes were not significantly expressed on the *Eucalyptus* tissue medium culture. This could be attributed to the destruction of the cuticle in the medium. However, one cutinase gene (Ca_Cap05169) in *C. pseudoreteaudii* was up-regulated > 225-fold early in leaf infection [49].

**Fig. 6:**
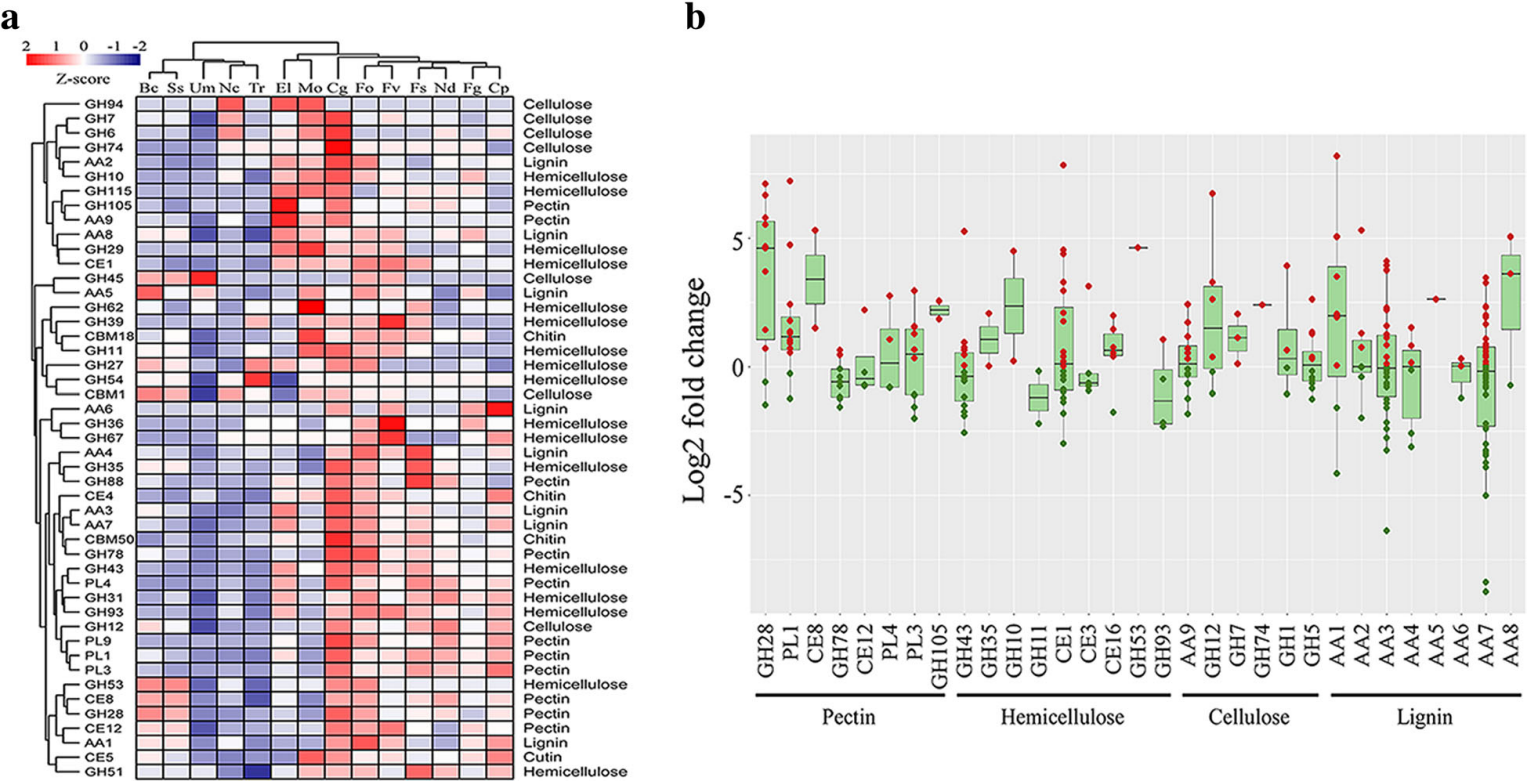
Cell wall degrading enzyme (CWDE) families in *C. pseudoreteaudii*. **a**. Hierarchical clustering of CWDE families from *C. pseudoreteaudii* and 13 fungal genomes. For fungi name abbreviations, see Fig. 2 and Fig. 4. Enzyme families are represented by their family number according to the carbohydrate-active enzyme database (http://www.cazy.org/). GH, glycoside hydrolase; CE, carbohydrate esterase; AA, auxiliary activities; CBM, carbohydrate-binding module; PL, polysaccharide lyase. Right side, known substrate of CWDE families. Overrepresented (white to red) and underrepresented (white to blue) domains are depicted as Z-scores for each family. Approximately unbiased (AU) P-values (%) are computed by 1000 bootstrap resamplings by using the R package pvclust. **b**. Plots of CWDE gene expression of each family in *Eucalyptus* tissue medium. Red circles, log2foldchange > 0; green circles, log2foldchange < 0. The substrates of CWDE families are indicated. The Tukey whiskers indicate 1.5 times the interquartile range from the 25th and 75th percentiles

*C. pseudoreteaudii* possesses more genes encoding the 1,4-benzoquinone reductase (family AA6) that function in the degradation of lignin and in the protection of fungal cells from reactive quinone compounds (Fig. 6a; Additional file 1: Table S7). In addition, *C. pseudoreteaudii* has more genes encoding multicopper oxidases (AA1) than most other ascomycetes pathogens. Most of these genes were up-regulated in *Eucalyptus* tissue medium culture (Fig. 5b), indicating a high potential for lignin-degradation.

Likewise, *C. pseudoreteaudii* had more pectin-degrading enzyme genes than other pathogens, including families GH28, PL3, PL11, and PL22 (Fig. 6a; Additional file 1: Table S7). Many of these pectinase genes were significantly up-regulated in *Eucalyptus* tissue medium culture. 12 genes of GH28 family were found in the *C. pseudoreteaudii* genome, many of which were up-regulated more than tenfold (Fig. 6b). Furthermore, one polygalacturonase gene (Ca_Cap14295) was up-regulated > 500-fold during the infection of *Eucalyptus* leaves [49]. These results imply that pectinase may play important role in the colonization of *C. pseudoreteaudii* on *Eucalyptus* leaves.

### *C. pseudoreteaudii* secretome is rich in potential virulence factors

Secreted proteins, particularly effectors, are essential for phytopathogens during their interactions with plants [24, 50, 51]. These proteins can degrade plant cell walls components or other substrates to facilitate the infection and the nourishment acquisition. They can also manipulate the environment of host cell to promote infection or elicit plant defense responses. In the current study, a total of 1178 secreted proteins were predicted in the genome of *C. pseudoreteaudii*, accounting for 8.2% of the proteome. These secreted proteins were significantly enriched in hydrolase activity, proteolysis, UDP-N-acetylmuramate dehydrogenase activity, cellulase activity, ferric iron binding, peroxidase activity, and cell wall macromolecule catabolic processes (Additional file 2: Figure S4).

Most of the identified pathogenic effectors are usually SSCPs. The disulfide bridges of partial cysteine residues can stabilize the structure and maintain the function of protein when transferred into the hostile environment of host cells [52]. Therefore, they play important roles in the compatible interaction with the host. In total, we found 207 SSCPs with lengths shorter than 300 amino acids and at least four cysteine residues in the mature proteins in *C.pseudoreteaudii* (Additional file 1: Table S8). Two SSCPs (Cp_Cap02912, Cp_Cap04435) had homologs with the LysM domain-containing proteins, which may play roles in the sequestration of chitin oligosaccharides and in dampening host defense [53]. Five of these SSCPs were up-regulated during infection of *Eucalyptus* [49].

## Conclusions

In this study, we sequenced the genome of *C. pseudoreteaudii*, a pathogen that is extensively distributed throughout southeast Asia. A 63.7 Mb genome with 14,355 coding genes were assembled. The genome size and coding capacity is similar to related species. The genome contains 9.26% repeat sequences, most of which are TEs. Comparative genomic analysis has led to the conclusion that *C. pseudoreteaudii* has evolved multiple strategies to adapt to the hostile ecological habitat of *Eucalyptus*.

*Eucalyptus* species have diverse and abundant secondary metabolites for defense against various pathogens. The recently released genome of *E. grandis* revealed that it has the largest observed number of terpene synthase gene among all sequenced plant genomes [54]. Furthermore, several phenylpropanoid gene families and a subgroup of R2R3-MYB transcription factor genes, known to be involved in the regulation of the phenylpropanoid pathway, are significantly expanded by tandem duplications. This indicates that *Eucalyptus* can produce a wide range of terpenoid and phenylpropanoid-derived compounds for defense. Thus, successful colonization of the pathogen in *Eucalyptus* leaves largely depends on the pathogen’s ability to metabolize or inactivate these phytoalexins. A striking feature of the *C.pseudoreteaudii* genome is the numerous genes that degrade secondary metabolites, such as tannase, (S)-2-hydroxy-acid oxidase, Cytochrome P450, and aromatic amino acid aminotransferases. Some transporter families relating with the removal of toxic compounds were observed to expand in the *C.pseudoreteaudii* genome. This suggested that *C. pseudoreteaudii* probably developed an effective detoxification system, including degradation and transportation to respond to the phytoalexin enriched in *Eucalyptus*.

*Fusarium* is a closely related genus to *C. pseudoreteaudii*, and employs a diversity of secondary metabolites as toxins during the host interaction. There are three types of mycotoxins: polyketides, including aurofusarin, fumonisin and zearalenone; terpenes, including trichothecene and carotenoid; and nonribosomal peptides, including siderophorethe [55]. These mycotoxins increase membrane permeability and lead to water loss in the host. They can also alter the cell’s organellar structures and function, influence enzyme activities, inhibit protein synthesis, and trigger PCD [56]. Toxin production is essential for some diseases to spread in hosts. For example, wheat and toxin sensitivity is positively correlated with wheat cultivar susceptibility and pathogenesis [57]. Surprisingly, an analysis of secondary metabolism gene clusters in the *C. pseudoreteaudii* genome revealed a significant expansion of secondary metabolites. Thus, it is likely that this fungal pathogen produces some secondary metabolites employed as toxins that lead to the characteristic symptoms of leaf blight. However, further research is necessary to elucidate the chemical nature and role of these putative secondary metabolites.

Compared to other pathogenic fungi, *C. pseudoreteaudii* harbors a large number of cutinase genes, indicative of a gene expansion to adapt to the host environment and facilitate plant cuticle degradation. This expansion of cutinase genes may be a reason that *C. pseudoreteaudii* can attack the resistant cultivars of *Eucalyptus* which have thicker cuticle. Further supporting this notion, genes involved in the degradation of pectin and lignin were also more than other pathogens, consistent with *C. pseudoreteaudii*’s ability to spread to the branch tissue and cause cutting rot.

## Methods

### Sequenced strain

*C. pseudoreteaudii* YA51 strain was originally isolated from a *Eucalyptus* tree with the typical symptoms of leaf blight [15]. The sample was deposited at the Forestry protection institute, Fujian Agriculture and Forestry University, Fuzhou, PR China (Deposited number: FAFUYA201105001). For genome sequencing, strain YA51 was cultured on PDA medium for 7 days, and then transferred to 150 mL PDB medium for 2 days. Mycelia were filtered through sterile gauze and lyophilized. Genomic DNA was extracted using SDS-CTAB and stored at − 80 °C.

### Genome sequencing and assembly

The genome of *C. pseudoreteaudii* was sequenced using Illumina Hiseq platforms at Beijing Novogene Bioinformatics Technology Co., Ltd. (Novogene, China). Three Illumina paired-end libraries were constructed with an insertion size of 500 bp, 2 kb, and 6 kb, respectively. Low-quality reads were filtered by Trimmomatic [58]. The high-quality reads were used for de novo assembly and scaffolding using SOAP denovo (version 1.05, http://soap.genomics.org.cn/soapdenovo.html). Gaps closure was performed using GapCloser v1.12 [59, 60]. The completeness of the *C. pseudoreteaudii* genome was evaluated using CEGMA [61]. Repeat sequences were identified and classified using RepeatModeler v1.07, RepeatProteinMask and RepeatMasker v4-0-3.

### Gene prediction and annotation

Gene structure was predicted using the PASA pipeline with a combination of ab-initio and RNA-Seq evidence based approaches [62]. For ab-initio predictions, Augustus, SNAP, Transdecoder and GeneMark-ES v2 were employed to predict coding genes [63–65]. The EVidenceModeler (EVM) was used to compute weighted consensus gene annotations based on ab-initio gene models and transcript evidence derived from the Cufflinks RNA-seq assemblies in this study [62]. Finally, PASA was used again to update the EVM consensus prediction.

All predicted genes were functionally annotated by their sequence similarity to genes and proteins in several databases. For this, we used the BLASTp (e-value cutoff of 1e-5) to align the gene models against various proteins databases: non-redundant (NR) database at NCBI, SwissProt-databases containing only manually curated proteins, uniref90 and uniref100-databases containing clustered sets of proteins from UniProt, Pfam-database of protein families and KEGG-database of metabolic pathways. GO analysis of protein sequences were conducted by Blast2GO [66]. GO enrichment was performed by ClueGO with *P* value < 0.05 [67].

### Phylogenomic tree construction

To construct the phylogenomic tree of *C. pseudoreteaudii* and 13 other ascomycota isolates, including *Botrytis cinerea, Colletotrichum gloeosporioides, Eutypa lata, F. graminearum, F. oxysporum, F. solani, F. verticillioides, Magnaporthe oryzae, Neonectria ditissima, Neurospora crassa, Sclerotinia sclerotiorum, Trichoderma reesei and Ustilago maydis*, 1032 single copy genes shared by all genomes were selected by orthofinder and aligned with mafft (mafft-linsi-anysymbol) [68, 69]. The phylogenomic tree was constructed using FastTree based on the alignments of single-copy ortholog families with approximately-maximum-likelihood model and bootstrap 100 [70].

### Gene family analysis

Gene family annotation for *C. pseudoreteaudii* and the other organisms was based on a pfam local database with hmmer version 3.1b1 [71, 72]. The comparison of gene families across organisms was conducted by CAFE with lambda 0.314, *P* value 0.01 and 1000 random samples [73].

Putative CAZymes were identified using the HMMER 3.1b1 with annotated HMM profiles of CAZymes downloaded from the dbCAN database [74]. The identification and classification of the membrane transporters superfamily were obtained by using blastp searches with e value <1e-5 and identity > 40% against the transporter protein database, downloaded from Transporter Classification Database [75]. The secretomes of 14 fungi in this study were identified by SignalP 4.1 and TMHMM 2.0 [76]. Core secondary metabolite (SM) genes and clusters were initially identified using antiSMASH. CYPs genes were identified with HMMER and then named using the cytochrome P450 homepage [77].

### Transcriptome analysis

*C. pseudoreteaudii* was cultured on PDB medium with 1% (*w*/*v*) *Eucalyptus* tissue (leaves of *E. grandis* ×*E. camaldulensis* M1 were ground into powder in liquid nitrogen using a mortar) for 2 days at 28 °C, 130 rpm. The mycelia were harvested with three biological replicates. Mycelia on PDB medium with no *Eucalyptus* tissue were used as control group.

Total RNA was extracted using RNAprep Pure Plant Kit (Tiangen Biotech CO., LTD). The quality and quantity of RNA were determined using a Nanodrop2000 (Thermo, Wilmington, USA) and Agilent 2100. Six libraries were constructed as previously reported at Beijing Novogene Bioinformatics Technology Co., Ltd. (Novogene, China). The insert sizes of all the libraries were 300 bp. They were sequenced with the Illumina HiSeq 2000 with 150 bp paired-end sequencing. All the clean reads were then mapped to the genome sequence of *C. pseudoreteaudii* using TopHat v 2.0.958 [78]. Gene expression levels were calculated using Cufflinks v2.0.266 based on the FPKM ([79]. Transcript with a significant *P* value (0.05) and a greater than two-fold change (log2) in transcript abundance was considered as differentially expressed gene.

## Additional files

Additional file 1: **Table S1.** Sequencing statistics of *C. pseudoreteaudii*. **Table S2.** Repeat sequences annotation of *C. pseudoreteaudii*. **Table S3.** Inventory of secondary metabolism backbone enzymes in *C. pseudoreteaudii*. **Table S4.** Inventory of secondary metabolism gene clusters in *C. pseudoreteaudii*. **Table S5.** Inventory of membrane transporters in *C. pseudoreteaudii*. **Table S6.** Upregulated (fold-change > 2) *C. pseudoreteaudii* membrane transporters in *Eucalytpus* tissue induced medium vs in PDB medium. **Table S7.** Number of carbohydrate-active enzyme modules of *C. pseudoreteaudii* and 13 other fungi according to the CAZy database. **Table S8.** Inventory of putative effectors in *C. pseudoreteaudii*. (XLS 238 kb)

Additional file 2: **Figure S1.** Evolutionary genealogy of genes: Non-supervised Orthologous Groups (eggNOG) function annotation of the *C. pseudoreteaudii* genome. In total, there are 11,760 genes (81.92%) that have functional assignments. **Figure S2.** Gene ontology (GO) functional classification of the *C. pseudoreteaudii* genome. In total, there are 8972 genes (62.5%) that have functional assignments. **Figure S3.** Overrepresented GO categories of gene specific in *C. pseudoreteaudii*. **Figure S4.** GO term enrichment analysis of the expanded gene families in *C. pseudoreteaudii*. **Figure S5.** GO functional annotation of differentially expressed genes of *C. pseudoreteaudii* in *Eucalyptus* tissue medium culture (log2 fold-changes). a. Up-regulated genes. b. Down-regulated genes.The X- axis represents the number of genes in a functional group. (ZIP 12374 kb)

## Abbreviations

AAs: Auxillary Activities
ABC: ATP-binding cassette
CAZymes: Carbohydrate-active enzymes
CBMs: Carbohydrate-Binding Modules
CE: Carbohydrate Esterases
CEGMA: Core Eukaryotic Gene Mapping Approach
CLBs: *Calonectria* leaf blight
CYPs: Cytochrome P450
DHA1: Drug: H+ Antiporter-1
DMAT: Dimethylallyl tryptophan synthases
FPKM: Fragments per kilobase of exon model per millions mapped reads
GH: Glycoside Hydrolases
GO: Gene Ontology
GT: Glycosyl Transferases
KEGG: Kyoto Encyclopaedia of Genes and Genomes
LSGR: Lineage-specific genomic regions
MFS: Multi-drug resistant transporters
NOG: Non-Supervised Orthologous Groups
NRPS: Nonribosomal peptide synthases
PCD: Programmed cell death
PDA: Potato dextrose agar
PDB: Potato dextrose broth
PKS: Polyketide synthases
PL: Polysaccharide Lyases
SDS-CTAB: Sodium dodecyl sulfate-hexadecyl trimethyl ammonium bromide
SITs: Siderophore-iron transporters
SSCPs: Cysteine-rich proteins
TEs: Transposable elements
