# Supplementary material for: Whole genome and transcriptome analysis reveal adaptive strategies and pathogenesis of Calonectria pseudoreteaudii to Eucalyptus
Source: BMC Genomics. 2018 May 10;19:358. doi: 10.1186/s12864-018-4739-1 (PMC5946483; doi:10.1186/s12864-018-4739-1)
Supplement: Supplementary file 2 — Figure S1. Evolutionary genealogy of genes: Non-supervised Orthologous Groups (eggNOG) function annotation of the C. pseudoreteaudii genome. In total, there are 11,760 genes (81.92%) that have functional assignments. Figure S2. Gene ontology (GO) functional classification of the C. pseudoreteaudii genome. In total, there are 8972 genes (62.5%) that have functional assignments. Figure S3. Overrepresented GO categories of gene specific in C. pseudoreteaudii. Figure S4. GO term enrichment analysis of the expanded gene families in C. pseudoreteaudii. Figure S5. GO functional annotation of differentially expressed genes of C. pseudoreteaudii in Eucalyptus tissue medium culture (log2 fold-changes). a. Up-regulated genes. b. Down-regulated genes.The X- axis represents the number of genes in a functional group. (ZIP 12374 kb) [file 12864_2018_4739_MOESM2_ESM.zip › Figure S1.pdf]

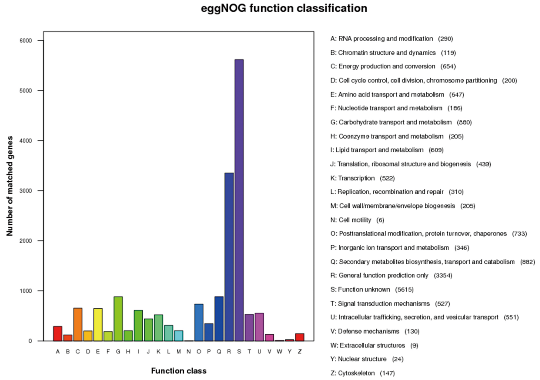
eggNOG function classification
Number of matched genes
0
1000
2000
3000
4000
5000
6000
A B C D E F G H I J K L M N O P Q R S T U V W Y Z
Function class
A: RNA processing and modification (290)
B: Chromatin structure and dynamics (119)
C: Energy production and conversion (654)
D: Cell cycle control, cell division, chromosome partitioning (200)
E: Amino acid transport and metabolism (647)
F: Nucleotide transport and metabolism (186)
G: Carbohydrate transport and metabolism (880)
H: Coenzyme transport and metabolism (205)
I: Lipid transport and metabolism (609)
J: Translation, ribosomal structure and biogenesis (439)
K: Transcription (522)
L: Replication, recombination and repair (310)
M: Cell wall/membrane/envelope biogenesis (205)
N: Cell motility (6)
O: Posttranslational modification, protein turnover, chaperones (733)
P: Inorganic ion transport and metabolism (346)
Q: Secondary metabolites biosynthesis, transport and catabolism (882)
R: General function prediction only (3354)
S: Function unknown (5615)
T: Signal transduction mechanisms (527)
U: Intracellular trafficking, secretion, and vesicular transport (551)
V: Defense mechanisms (130)
W: Extracellular structures (9)
Y: Nuclear structure (24)
Z: Cytoskeleton (147)
