# Supplementary figures and images for: Whole genome and transcriptome analysis reveal adaptive strategies and pathogenesis of Calonectria pseudoreteaudii to Eucalyptus
Source: BMC Genomics. 2018 May 10;19:358. doi: 10.1186/s12864-018-4739-1 (PMC5946483; doi:10.1186/s12864-018-4739-1)

# Go Standard

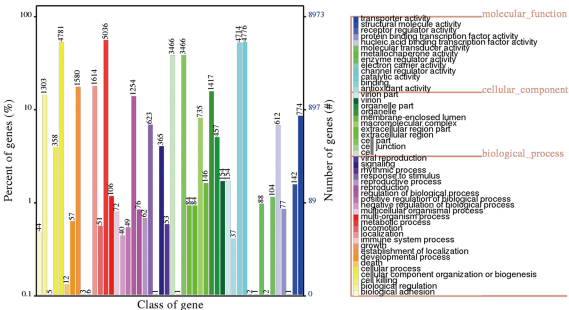

Supplement: Supplementary file 2 — Figure S1. Evolutionary genealogy of genes: Non-supervised Orthologous Groups (eggNOG) function annotation of the C. pseudoreteaudii genome. In total, there are 11,760 genes (81.92%) that have functional assignments. Figure S2. Gene ontology (GO) functional classification of the C. pseudoreteaudii genome. In total, there are 8972 genes (62.5%) that have functional assignments. Figure S3. Overrepresented GO categories of gene specific in C. pseudoreteaudii. Figure S4. GO term enrichment analysis of the expanded gene families in C. pseudoreteaudii. Figure S5. GO functional annotation of differentially expressed genes of C. pseudoreteaudii in Eucalyptus tissue medium culture (log2 fold-changes). a. Up-regulated genes. b. Down-regulated genes.The X- axis represents the number of genes in a functional group. (ZIP 12374 kb) [file 12864_2018_4739_MOESM2_ESM.zip › Figure S2.pdf]

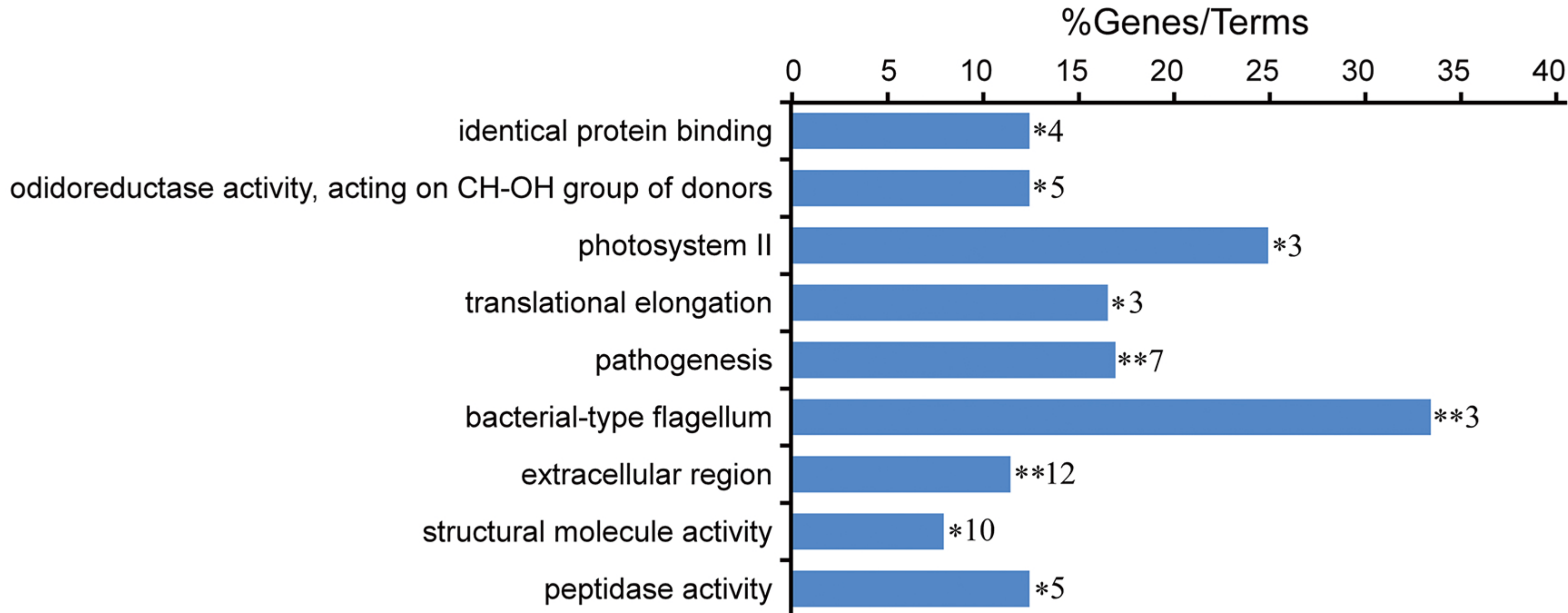

Supplement: Supplementary file 2 — Figure S1. Evolutionary genealogy of genes: Non-supervised Orthologous Groups (eggNOG) function annotation of the C. pseudoreteaudii genome. In total, there are 11,760 genes (81.92%) that have functional assignments. Figure S2. Gene ontology (GO) functional classification of the C. pseudoreteaudii genome. In total, there are 8972 genes (62.5%) that have functional assignments. Figure S3. Overrepresented GO categories of gene specific in C. pseudoreteaudii. Figure S4. GO term enrichment analysis of the expanded gene families in C. pseudoreteaudii. Figure S5. GO functional annotation of differentially expressed genes of C. pseudoreteaudii in Eucalyptus tissue medium culture (log2 fold-changes). a. Up-regulated genes. b. Down-regulated genes.The X- axis represents the number of genes in a functional group. (ZIP 12374 kb) [file 12864_2018_4739_MOESM2_ESM.zip › Figure S3.pdf]

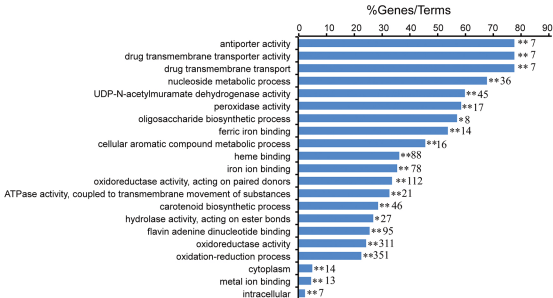

Supplement: Supplementary file 2 — Figure S1. Evolutionary genealogy of genes: Non-supervised Orthologous Groups (eggNOG) function annotation of the C. pseudoreteaudii genome. In total, there are 11,760 genes (81.92%) that have functional assignments. Figure S2. Gene ontology (GO) functional classification of the C. pseudoreteaudii genome. In total, there are 8972 genes (62.5%) that have functional assignments. Figure S3. Overrepresented GO categories of gene specific in C. pseudoreteaudii. Figure S4. GO term enrichment analysis of the expanded gene families in C. pseudoreteaudii. Figure S5. GO functional annotation of differentially expressed genes of C. pseudoreteaudii in Eucalyptus tissue medium culture (log2 fold-changes). a. Up-regulated genes. b. Down-regulated genes.The X- axis represents the number of genes in a functional group. (ZIP 12374 kb) [file 12864_2018_4739_MOESM2_ESM.zip › Figure S4.pdf]

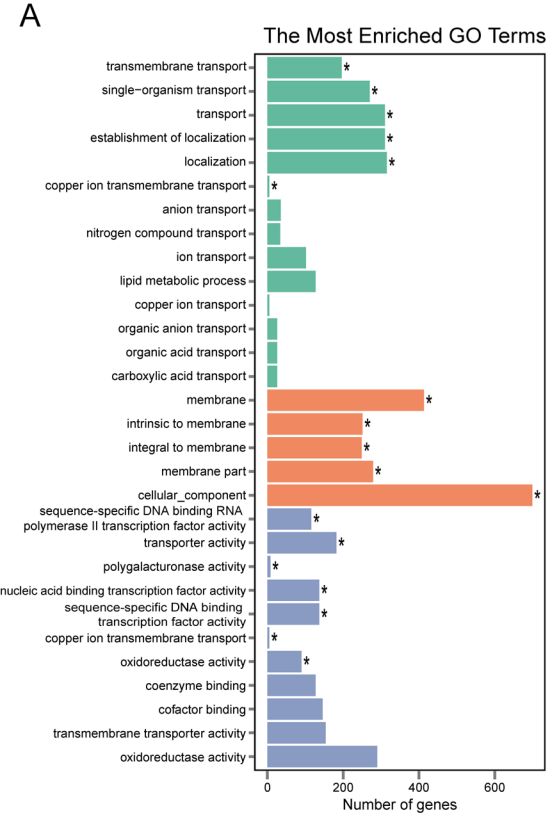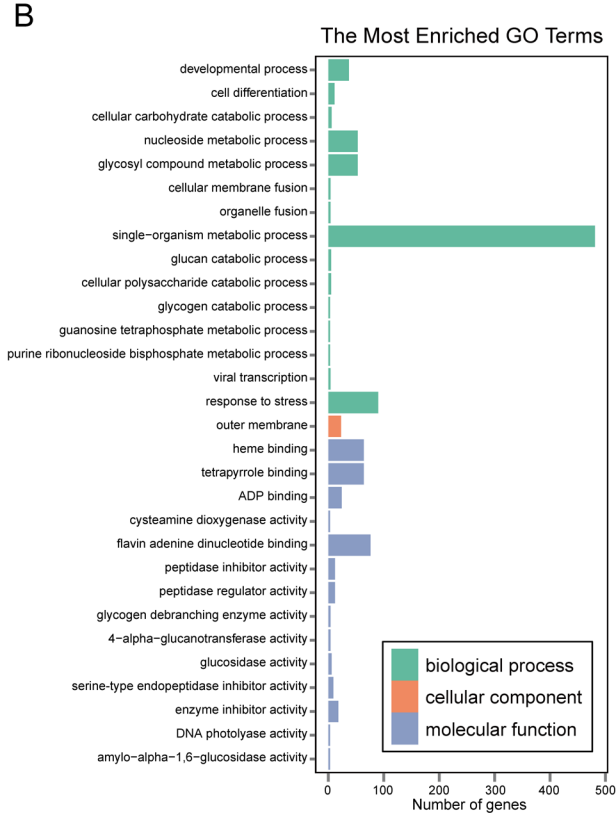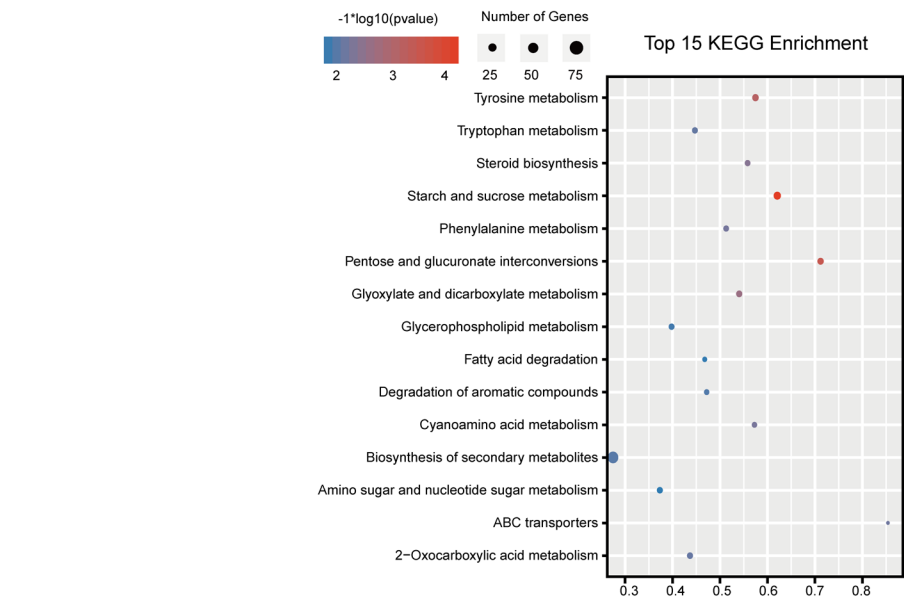

Supplement: Supplementary file 2 — Figure S1. Evolutionary genealogy of genes: Non-supervised Orthologous Groups (eggNOG) function annotation of the C. pseudoreteaudii genome. In total, there are 11,760 genes (81.92%) that have functional assignments. Figure S2. Gene ontology (GO) functional classification of the C. pseudoreteaudii genome. In total, there are 8972 genes (62.5%) that have functional assignments. Figure S3. Overrepresented GO categories of gene specific in C. pseudoreteaudii. Figure S4. GO term enrichment analysis of the expanded gene families in C. pseudoreteaudii. Figure S5. GO functional annotation of differentially expressed genes of C. pseudoreteaudii in Eucalyptus tissue medium culture (log2 fold-changes). a. Up-regulated genes. b. Down-regulated genes.The X- axis represents the number of genes in a functional group. (ZIP 12374 kb) [file 12864_2018_4739_MOESM2_ESM.zip › Figure S5.pdf]
